# Supplementary material for: The role of Cdx2 as a lineage specific transcriptional repressor for pluripotent network during the first developmental cell lineage segregation
Source: Sci Rep. 2017 Dec 7;7:17156. doi: 10.1038/s41598-017-16009-w (PMC5719399; doi:10.1038/s41598-017-16009-w)
Supplement: Supplementary file 5 — Supplementary Figures [file 41598_2017_16009_MOESM5_ESM.zip › Supplementary Figures.pdf]

The role of Cdx2 as a lineage specific transcriptional repressor for pluripotent network during the first developmental cell lineage segregation

Authors: Daosheng Huang, Xiaoping Han, Ping Yuan, Amy Ralston, Lingang Sun, Mikael Huss, Tapan Mistri, Luca Pinello, Huck Hui Ng, Guocheng Yuan, Junfeng Ji, Janet Rossant, Paul Robson & Guoji Guo

Supplementary Materials

Figure S1

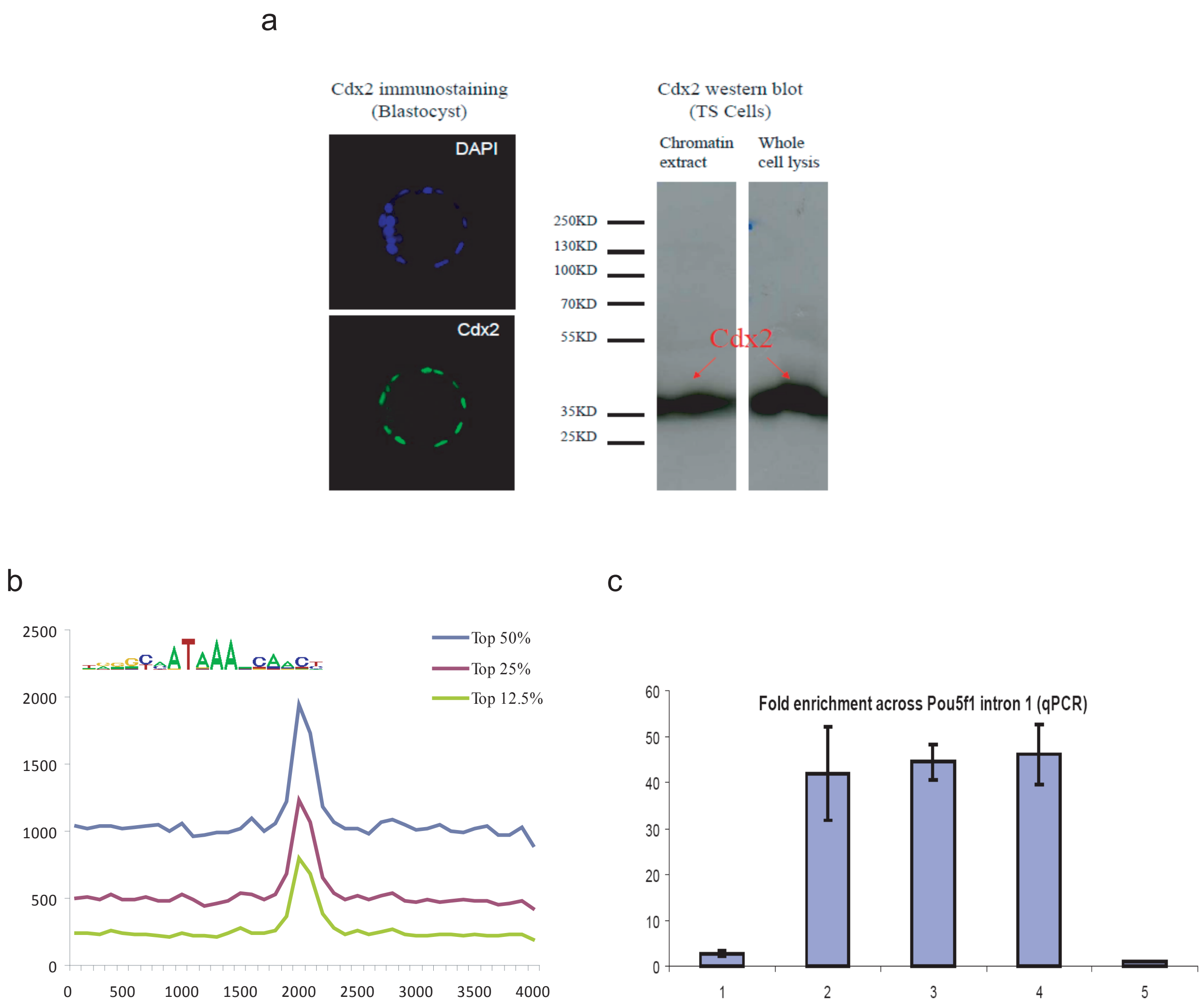

Figure S1 CDX2 ChIP-Seq in trophoblast stem cells (Related to Figure 3)

(a) Immunostaining assay in blastocyst and western blotting in TS cell show high specificity of CDX2 antibody.

(b) CDX2 motif is overrepresented in ChIP-enriched regions, as we looked at motif counts across 4kb windows

(c) ChIP-qPCR results show that CDX2 binds to *Pou5f1* intron with more than 40 fold enrichment across the binding region

Figure S2

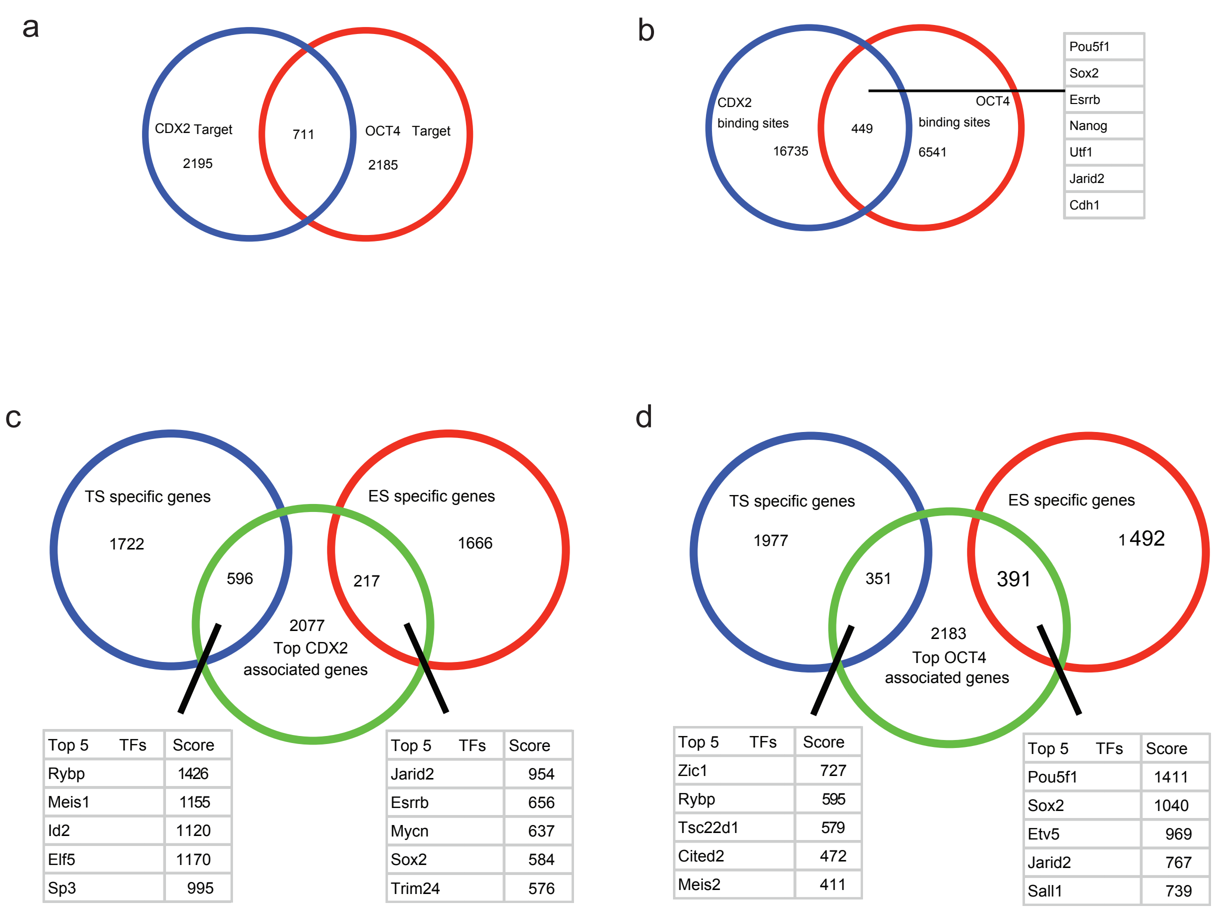

Top 5 TFs	Score
Rybp	1426
Meis1	1155
Id2	1120
Elf5	1170
Sp3	995

Top 5 TFs	Score
Jarid2	954
Esrrb	656
Mycn	637
Sox2	584
Trim24	576

Top 5 TFs	Score
Zic1	727
Rybp	595
Tsc22d1	579
Cited2	472
Meis2	411

Top 5 TFs	Score
Pou5f1	1411
Sox2	1040
Etv5	969
Jarid2	767
Sall1	739

Figure S2 Comparison of CDX2 targets and OCT4 targets(Related to Figure 4)

(a) Venn diagram shows that OCT4 and CDX2 have a significant overlap in downstream targets
(b) Venn diagram shows that OCT4 and CDX2 have a significant overlap in binding site
(c) Combination of CDX2 bindings with its function in TS cells and ES cells .we overlap our defined top 3000 CDX2 binding targets with TS-specific gene and ES-specific gene.
(d) Combination of OCT4 bindings with its function in TS cells and ES cells .we overlap our defined top 3000 OCT4 binding targets with TS-specific gene and ES-specific gene.

Figure S3

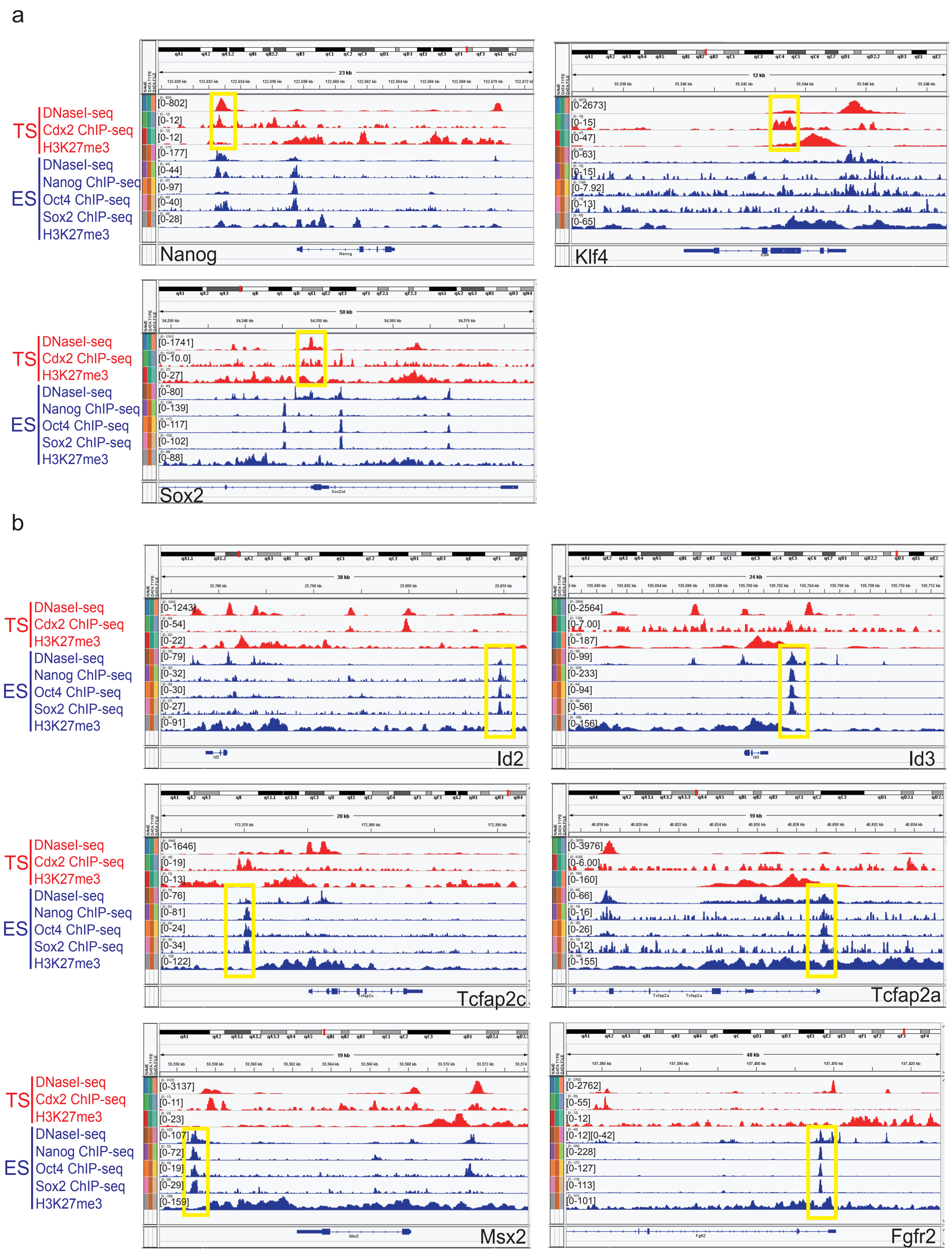

Figure S3 Integrated binding pattern of key lineage-specific genes in ES and TS cells. (Related to Figure 4)

(a) Cdx2 binding sites at ES pluripotent markers are enriched with DNase I hypersensitive and H3K27me3 signals, includes *Nanog*, *Sox2* and *Klf4*.

(b) OSN binding sites at TS specific markers are enriched with DNase I hypersensitive and H3K27me3 signals, includes *Id2, Id3, Tcfap2a, Tcfap2c, Msx2* and *Fgfr2*.

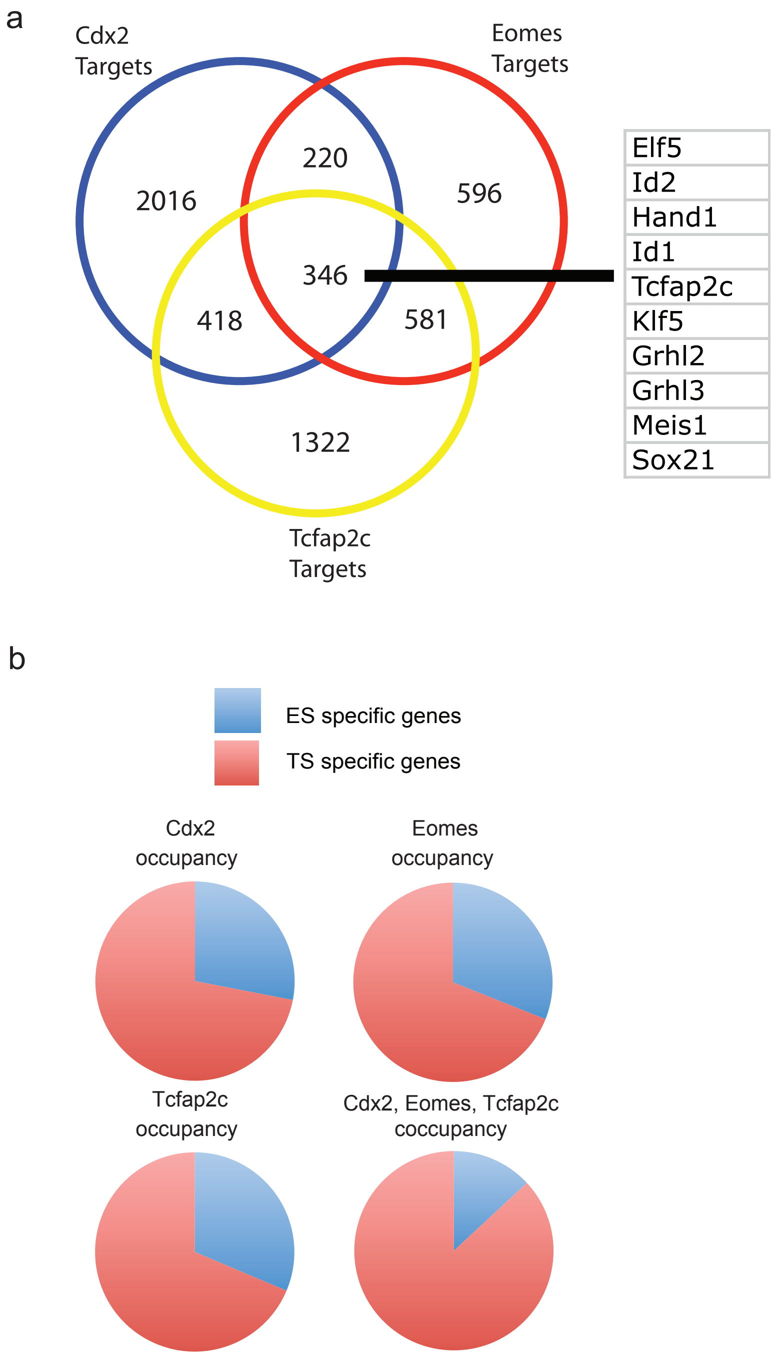

Figure S4 Integration analysis of Core regulators in TS cells.

(a) Cdx2 co-regulate important TE lineage markers with Eomes and Tcfap2c. Top Cdx2 ChIP-seq associated transcription factors within the Cdx2, Eomes and Tcfap2c co-target list are shown in the table.

(b) Cdx2, Eomes and Tcfap2c co-targets are more likely to be activation targets (TS lineage specific) rather than repression targets (ES lineage specific).
